# Hepatocyte growth factor gene therapy enhances infiltration of macrophages and may induce kidney repair in *db*/*db* mice as a model of diabetes

**DOI:** 10.1007/s00125-012-2535-z

**Published:** 2012-03-30

**Authors:** M. Flaquer, M. Franquesa, A. Vidal, N. Bolaños, J. Torras, N. Lloberas, I. Herrero-Fresneda, J. M. Grinyó, J. M. Cruzado

**Affiliations:** 1 Nephrology Laboratory, Departament de Ciències Clíniques, IDIBELL, University of Barcelona, Bellvitge Hospital, Barcelona, Spain; 2 Pathology Service, IDIBELL, Bellvitge Hospital, University of Barcelona, Barcelona, Spain; 3 Servei de Nefrologia, Department of Nephrology, Hospital Universitari de Bellvitge, University of Barcelona, IDIBELL, Feixa Llarga s/n, L’Hospitalet de Llobregat, 08907 Barcelona, Spain

**Keywords:** Bone marrow stem cells, Diabetic nephropathy, *HGF* gene therapy, Macrophages, Renal repair

## Abstract

**Aims/hypothesis:**

We previously demonstrated hepatocyte growth factor (HGF) gene therapy was able to induce regression of glomerulosclerosis in diabetic nephropathy through local reparative mechanisms. The aim of this study was to test whether bone-marow-derived cells are also involved in this HGF-induced reparative process.

**Methods:**

We have created chimeric *db/db* mice as a model of diabetes that produce enhanced green fluorescent protein (EGFP) in bone marrow cells. We performed treatment with *HGF* gene therapy either alone or in combination with granulocyte-colony stimulating factor, in order to induce mobilisation of haematopoietic stem cells in these diabetic and chimeric animals.

**Results:**

We find *HGF* gene therapy enhances renal expression of stromal-cell-derived factor-1 and is subsequently associated with an increased number of bone-marrow-derived cells getting into the injured kidneys. These cells are mainly monocyte-derived macrophages, which may contribute to the renal tissue repair and regeneration consistently observed in our model. Finally, *HGF* gene therapy is associated with the presence of a small number of Bowman’s capsule parietal epithelial cells producing EGFP, suggesting they are fused with bone-marrow-derived cells and are contributing to podocyte repopulation.

**Conclusions/interpretation:**

Altogether, our findings provide new evidence about the therapeutic role of HGF and open new opportunities for inducing renal regeneration in diabetic nephropathy.

## Introduction

Chronic kidney disease (CKD) is a major health problem worldwide [1]. CKD is a risk factor for cardiovascular diseases and mortality. Diabetic nephropathy is the leading cause of CKD in developed countries [2]. Diabetic nephropathy is characterised by renal hypertrophy, glomerulosclerosis, arteriolar hyalinosis and tubulo-interstitial fibrosis [3]. As in other diseases evolving to renal scarring, growth factors such as TGF-β1 and connective tissue growth factor (CTGF) are involved in the progression of renal damage [4]. There are very few therapies that induce renal repair in chronic nephropathies [5]. Hepatocyte growth factor (HGF) is a mesenchyme-derived cytokine with anti-fibrotic and regenerative properties in some experimental models of chronic renal damage [6, 7]. HGF inhibition exacerbates renal fibrosis, whereas HGF supplementation reverses progression [8–11]. We have previously studied the effect of *HGF* gene therapy on diabetic kidney disease in rats and have demonstrated HGF-supplementation-induced regression of glomerular sclerosis [12]. The reparative role of HGF has been robustly recognised in many studies [11]. The majority of HGF-established reparative mechanisms are directly related to a local effect of TGF-β1 inhibition [13], by increasing extracellular matrix degradation [14] or by inhibiting epithelial to mesenchymal transition [15]. However, there is a lack of information about the potential role of bone-marrow-derived cells on the regression of CKD induced by HGF. Kollet et al [16] demonstrated that, after liver injury, HGF facilitated bone marrow haematopoietic stem cell (HSC) motility, *CXCR4* expression and stromal-cell-derived factor-1 (SDF-1)-mediated directional migration of these cells to the damaged liver. More recently, Higashiyama et al [17] studied spontaneous liver regeneration after cirrhosis in wild-type and transgenic animals overexpressing *Hgf*. Animals overexpressing *Hgf* healed more rapidly because resident as well as bone-marrow-derived cells infiltrating the liver were producing some metalloproteases that mainly contributed to regression of liver fibrosis.

In order to study whether bone-marrow-derived cells are involved in the reparative mechanisms of HGF on diabetic kidney disease, we have created chimeric diabetic *db/db* mice. We performed bone marrow transplantation (BMT) using transgenic C57BL/6J mice producing the enhanced green fluorescent protein (EGFP) as donors to *db*/*db* mouse recipients.

## Methods

### Animals

The experiments complied with current legislation on animal experiments in the European Union, and the principles of laboratory animal care and were approved by our institution’s Ethics Committee for Animal Research. Female C57BLKS mice (*db*/*db*), 8 weeks old, were purchased from Janvier (Laval, France). Transgenic C57BL/6J mice producing EGFP were kindly provided by J. Barquinero (Unitat de Diagnòstic i Teràpia Molecular, Centre de Transfusió i Banc de Teixits, Barcelona, Spain).

### Bone marrow transplantation

#### Bone marrow isolation

In a sterile environment, bone marrow cells were isolated from the femurs and tibias of C57BL/6J EGFP^+^ mice, by flushing the bones with Iscove’s modified Dulbecco’s Medium (Invitrogen, Madrid, Spain) supplemented with 10% (vol./vol.) FBS, penicillin/streptomycin and l-glutamine. Samples were passed through a 70 μm cell strainer (BD Biosciences, Aalst, Belgium) to eliminate debris and break up cell clumps. After washing twice, cells were resuspended in PBS and stored at 4°C.

#### Irradiation and bone marrow transplantation

We performed BMT from EGFP^+^ C57BL/6J donor mice. Recipients were obese mice C57BLKS (*db*/*db*) as a model of diabetes. These animals were transplanted at 24 weeks of age, an age at which diabetic nephropathy is already established. Recipient *db*/*db* mice were lethally irradiated with a dose of 10.5 Gy and reconstituted 1 h post irradiation with 7.5 × 10^6^ donor cells/animal by intravenous injection in the tail vein, as previously described [18]. After BMT, transplanted animals were placed in cages in a sterile environment for 10 days.

#### Chimerism analysis

Chimerism was analysed 5 weeks after BMT. Peripheral blood was extracted from the tail vein. Blood was collected in Microvette CB300 with EDTA tubes (Sarstedt, Madrid, Spain). A 50 μl blood sample was mixed with 1 ml lysis buffer (BD Biosciences, San Jose, CA, USA) in order to discard red cells and the mixture was then incubated at 37°C for 3 min. Samples containing peripheral blood mononuclear cells (PBMCs) were washed twice (200 *g*, 5 min), stained with a viability marker, 7-amino-actinomycin D (BD Biosciences, San Jose, CA, USA), in order to distinguish living cells from dead cells, and were finally analysed by BD FACSCanto II (BD Biosciences, San Jose, CA, USA). Animals were considered chimeric when the percentage of EGFP^+^ cells was higher than 30% of the total PBMCs.

### Study groups

Chimeric animals were divided into four treatment groups and followed for 4 weeks: (1) *db*/*db*–BMT (*n* = 11), diabetic animals with BMT; (2) *db*/*db*+*HGF* (*n* = 12), diabetic animals with BMT and treated by *HGF*; (3) *db*/*db*+granulocyte-colony stimulating factor (G-CSF) (*n* = 10), diabetic animals with BMT and treated by G-CSF and (4) *db*/*db*+*HGF*+G-CSF (*n* = 11), diabetic animals with BMT and treated by *HGF* and G-CSF. We used *db* ^/−^ (*n* = 10), non-diabetic animals, and *db*/*db* (*n* = 10), diabetic animals, as age-matched control groups.

### Therapeutic interventions

#### HGF gene therapy

The *HGF* expression vector used in the present study was constructed and optimised as previously described by our group [19]. Two hours before *HGF* administration, 25 U of hyaluronidase (Sigma, Seelze, Germany) diluted in 60 μl of NaCl was injected into the muscle in order to improve plasmid DNA diffusion. Then, 50 μl of *HGF* (2 μg/μl) was injected into each leg. After the *HGF* intramuscular injection, the area around the injection was electroporated. The electric pulses were applied to the muscle by pulse generator (BTX ECM830 electroporator; Genetronics, San Diego, CA, USA) [19]. Six pulses of 100 ms, each with a voltage of 25 V, were applied. This protocol was performed twice, at days 3 and 17. Human HGF levels were analysed by Quantikine Human HGF immunoassay (R&D Systems, Oxford, UK) in order to assess the efficiency of gene therapy.

#### G-CSF administration

G-CSF (Filgrastim, Amgen, Thousand Oaks, CA, USA), 300 μg kg^−1^ day^−1^, was intraperitoneally administrated for 12 days in two periods, from days 1 to 6 and from days 15 to 20. Leucocyte counts were analysed to assess the response to G-CSF.

### Monitoring

Animals were followed from 8 to 33 weeks of age. During this period glucose levels and body weight were measured weekly (glucose was measured using the Glucocard G+meter GT-1820 [Menarini, Barcelona, Spain]). BMT was performed when animals were 24 weeks old. Chimerism was analysed 5 weeks after BMT, and the animals were killed in week 33. Urine and blood samples were collected in order to analyse urinary albumin and serum creatinine concentrations at three time points: before BMT (24 weeks of age), when chimerism was analysed (29 weeks of age) and before the mice were killed (33 weeks of age).

Mice were placed in metabolism cages in order to collect 24 h urine specimens before BMT (week 24), and thereafter before and after therapeutic interventions (at weeks 29 and 33, respectively). Blood was obtained from the tail vein. Serum creatinine and urine creatinine were determined by an autoanalyser (Olympus AU400; Olympus, Hamburg, Germany). Urine albumin excretion was determined using a specific commercially available ELISA kit (Albumin Blue Fluorescent Assay Kit; Active Motif, La Hulpe, Brussels, Belgium).

### Circulating haematopoietic stem cells

Analysis of HSCs was performed using a BD FACSCanto II (BD Biosciences, San Jose, CA, USA). Peripheral blood was collected at baseline and then 2 and 5 days after each *HGF* administration. Whole blood was incubated with allophycocyanin-conjugated anti-lineage (Lin) cocktail, phycoerythrin (PE)-Cy7-conjugated anti-cKit (also known as CD117) and PE-conjugated anti-stem cell antigen (Sca)-1 antibodies (BD Biosciences, Aalst, Belgium). Blood was then incubated with lysis buffer in order to discard erythrocytes. PBMCs were finally incubated with 7-amino-actinomycin D for dead cell staining. PBMCs were considered HSCs when they were Lin^−^ cKit^+^ Sca-1^+^ as previously described [20].

### Optical microscopy, immunohistochemical, immunofluorescence and confocal studies

Renal slices were fixed in 10% (vol./vol.) formalin and embedded in paraffin. Histological cross sections of 3 μm thickness were stained with haematoxylin and eosin, periodic acid–Schiff and Masson’s trichrome for optical microscopy assessment. Fibronectin (1:500) and SDF-1 (1:25) (Abcam, Madrid, Spain) were stained using the immunohistochemical technique described previously [21]. Anti-collagen IV (1:100; Millipore, Livingston, UK), anti-green fluorescent protein (GFP; 1:500, Abcam), anti-α-smooth muscle actin (SMA) (1:100, Sigma-Aldrich, Madrid, Spain), anti-claudin-I (1:19, Invitrogen, Paisley, UK), anti-WT-1 (1:30, Santa Cruz Biotechnology, Heidelberg, Germany), anti-F4/80 (1:50, LabClinics, Barcelona, Spain), anti-galectin-3 (1:100, Abcam) and anti-mannose-receptor (1:50, Abcam) antibodies were analysed using immunofluorescence. Slides were stained with: collagen IV and fibronectin to assess glomerulosclerosis; α-SMA to stain mesangial cells; claudin-I for epithelial cells from the Bowman’s capsule; WT-1 for podocytes; F4/80 for macrophages; CD90, CD73, CD105 for mesenchymal cells; CD34 for endothelial cells; and galectin-3 and mannose receptor for the M2 macrophage subpopulation.

Double immunolabelling was performed using anti-GFP with anti-α-SMA, anti-claudin-I, anti-WT-1 or anti-F4/80 antibodies; and anti-F4/80 with anti-galectin-3 and anti-mannose receptor. Optical microscopy, fibronectin, SDF-1 and collagen IV were evaluated (from 0 to +3) by a pathologist (A. Vidal) who was blinded to group assignment. Macrophages and the M2 subpopulation were evaluated in ten glomeruli for every sample in a blinded manner. Macrophage counts were expressed as macrophage/glomeruli. The percentage of M2^+^ with respect to F4/80+ macrophages was calculated (*n* = 4 per group).

Additional renal slices were embedded and frozen in optimal cutting temperature compound. Subsequently, cross sections of 5 μm thickness were fixed with 4% (wt/vol.) paraformaldehyde at 4°C for 20 min and incubated with the following primary antibodies: anti-CD90; anti-CD73; anti-CD105; and anti-CD34 (1:50, BD Pharmingen, Aalst, Belgium). Also, the amount of EGFP in renal frozen tissue was directly viewed with microscopy and automatically quantified using Leica Confocal Software. Finally, confocal studies (using a Leica TCS-SL confocal espectral microscope [Mannheim, Germany]) were performed in a blinded manner on paraffin-embedded and frozen renal tissues. Alexa Fluor647 (far red), Alexa Fluor488 (green), Alexa Fluor546 (red) and Alexa Fluor555 (red) were used as secondary antibodies (Molecular Probes, Invitrogen, Madrid, Spain). In a blinded manner, podocytes per glomerular tuft were quantified in ten glomeruli per sample and calculated as podocyte/total nuclei ratio. Nuclei were stained blue with DRAQ5 and counted using Fiji Is Just ImageJ software (http://fiji.sc).

### Gene expression analysis

RNA was extracted from kidney with PureLink RNA Mini Kit (Invitrogen, Madrid, Spain), using a Trizol reagent (Invitrogen, Madrid, Spain) to lyse the tissues, chloroform to separate the aqueous phase and organic phase (where the RNA remains), and ethanol to purify the RNA. RNA purity was analysed on a NanoDrop (NanoDrop ND-1000V3.3, Wilmington, DE, USA) and was considered pure when the absorbance ratio 260/280 nm was lower than 1.75. A total amount of 1,000 ng RNA was used to perform the reverse transcription using a High-Capacity cDNA Reverse Transcription Kit (Applied Biosystems, Warrington, UK). Thermal cycling conditions were 10 min at 25°C, 120 min at 37°C, 5 min at 85°C and finally held at 4°C. Tissue expression levels of *HGF*, *TGFβ* (also known as *TGFB1*) and *CTGF* were quantified by TaqMan real-time PCR (ABI Prism 7700, Applied Biosystems) using the comparative C_t_ method (Applied Biosystems).

### Cytokine analysis

Serum cytokines were quantitatively measured by FACSCanto with two different cytometric bead array (CBA) kits: the CBA mouse inflammation kit (IL-6, IL-10, MCP-1, IFN-γ, TNF and IL-12p70) and the CBA mouse Th1/Th2 cytokine kit (IL-4, IL-5, IFN-γ and TNF), both from BD Biosciences (San Jose, CA, USA). Data were acquired and analysed using BD CBA software (San Jose, CA, USA). If a sample had a cytokine concentration below the detection limit of the assay, a value of 0 was assigned for that particular cytokine concentration.

### Statistical analyses

All data are presented as mean±SE. Group means were compared with either the Student’s *t* test or ANOVA for parametric values, or the Mann–Whitney *U* test or Kruskal–Wallis test for non-parametric values. All *p* values were two-tailed, and a *p* value of less than 0.05 was considered statistically significant. All statistical analyses were analysed using StatView software.

## Results

### Bone marrow transplantation does not modify features of diabetic nephropathy in *db*/*db* mice

**Fig. 1:**
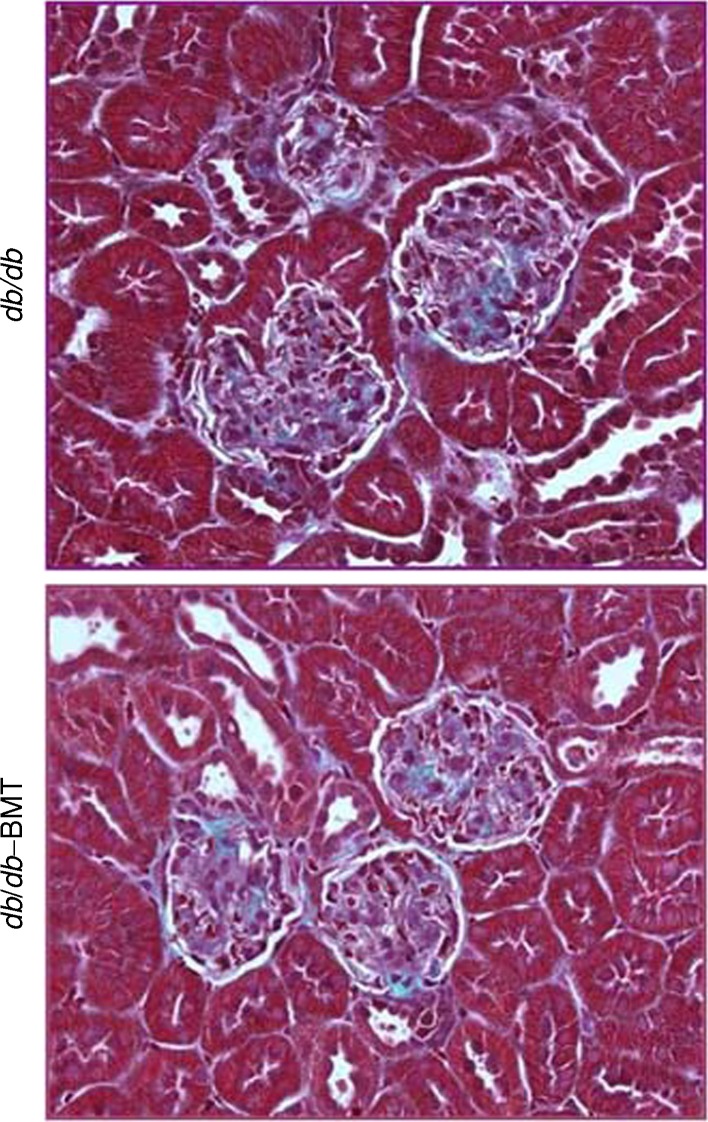
Glomerular sclerosis in *db*/*db*–BMT and *db*/*db* mice. Representative Masson’s trichrome-stained glomeruli showing similar glomerular sclerotic lesions. Magnification ×100

As we performed the experiments in EGFP^+^ chimeric mice, we first investigated whether BMT modified diabetic nephropathy in *db*/*db* mice. As shown in Table 1 and Fig. 1, both diabetic groups (*db*/*db* and *db*/*db*–BMT) were significantly different from the non-diabetic *db* ^/−^ group for body weight and albuminuria, as well as hyperglycaemia and glomerulosclerosis. There were no major differences between *db*/*db* and *db*/*db*–BMT. However, body weight was lower in the *db*/*db*–BMT mice compared with the *db*/*db* group, as would be expected as irradiation is usually associated with loss of body weight [22].

**Table 1:** Baseline and post-intervention variables associated with diabetic kidney disease

Characteristic	*db* ^/−^	*db*/*db*	*db*/*db*–BMT	*db*/*db*+*HGF*	*db*/*db*+G-CSF	*db*/*db*+*HGF*+G-CSF	*p*
Week 29 (pre-treatment)							
Body weight (g)	26.4 ± 0.8	32.8 ± 3	29.5 ± 2.5	34.0 ± 1.7*	34.4 ± 2.3*	31.4 ± 2.2	0.12
Glycaemia (mmol/l)	6 ± 0.16	33 ± 0.64*	30 ± 1.68*	23 ± 2.24*	27 ± 2.55*	26 ± 1.35*	<0.0001
Albuminuria (μg/24 h)	29 ± 4.5	535 ± 107.7*	470.9 ± 105.1*	456 ± 68.2*	425 ± 108.5*	450.4 ± 78.8*	0.002
Diuresis (ml)	0.6 ± 0.2	7 ± 0.9*	6.7 ± 1.1*	5.3 ± 1*	5.2 ± 1.4*	4.2 ± 1*	0.0043
Week 33 (post-treatment)							
Body weight (g)	27.6 ± 0.9	32.4 ± 3.3	27.1 ± 2.4	30.1 ± 1.5	28.4 ± 2.2	27.8 ± 2.3	0.56
Glycaemia (mmol/l)	6 ± 0.21	33 ± 0.05*	28 ± 2.51*	25 ± 2*^,†^	26 ± 2.85*^,†^	26 ± 1.35*^,†^	<0.0001
Albuminuria (μg/24 h)	23.2 ± 6.1	851 ± 188.8*	765.9 ± 165.5*	499 ± 251*	487 ± 115.4*	303 ± 48*	0.03
Diuresis (ml)	0.6 ± 0.1	27 ± 1.1*	12.5 ± 3*	5.6 ± 1.1	4.4 ± 1	5.4 ± 1.3	0.0001

*p < 0.05 vs *db* ^/−^; ^†^ *p* < 0.05 vs *db*/*db*; ANOVA followed by post hoc Fisher’s test

### HGF is renoprotective in chimeric *db*/*db* mice

**Fig. 2:**
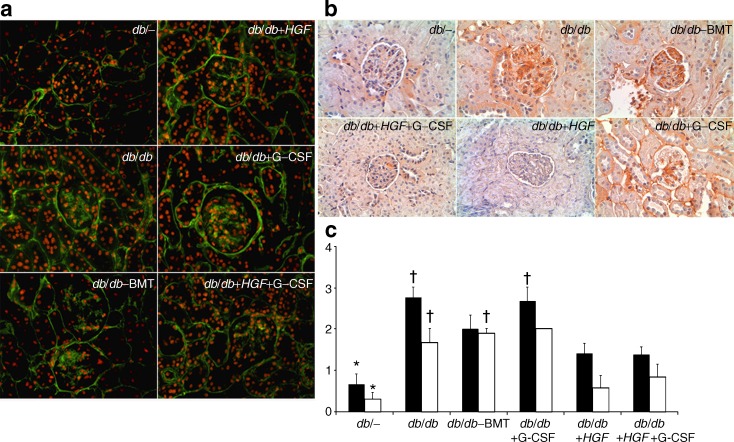
Effect of *HGF* gene therapy on diabetic kidney disease. **a** Collagen IV immunofluorescence (×630). Only *HGF* gene therapy (either alone or in combination with G-CSF) was associated with a significant decrease in collagen IV. **b** Fibronectin immunohistochemistry (×630). Groups treated with *HGF* therapy (*db*/*db*+*HGF* and *db*/*db*+*HGF*+G-CSF) showed decreased fibronectin accumulation. **c** Histogram summarising the mean values of collagen IV and fibronectin from each group. Black bars, collagen IV; white bars, fibronectin *p < 0.05 vs *db*/*db*, *db*/*db*–BMT and *db*/*db*+G-CSF; ^†^ *p* < 0.05 vs *db*/*db*+*HGF* and *db*/*db*+*HGF*+G-CSF (Kruskal–Wallis and Mann–Whitney *U* tests)

**Fig. 3:**
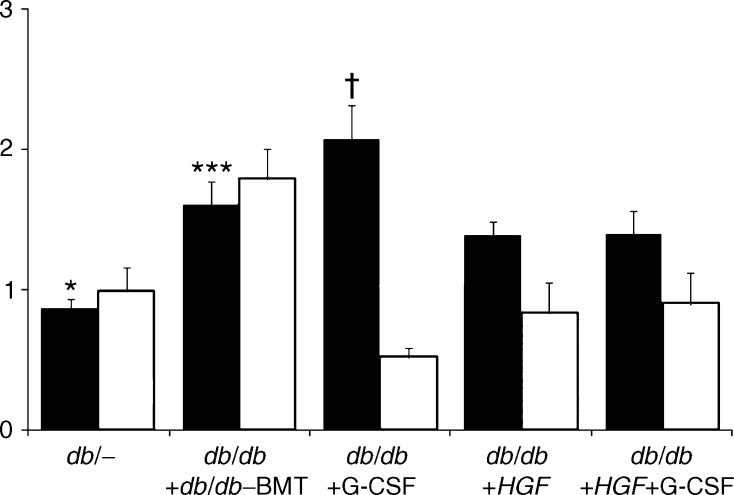
Effect of *HGF* gene therapy on *TGFβ1*/*HGF* ratio and *CTGF* gene expression in renal tissue. Mean values of *TGFβ*/*HGF* ratio and *CTGF*. Black bars, *TGFβ*/*HGF*; white bars, *CTGF*. *p < 0.05 vs *db*/*db*+*db*/*db*–BMT and *db*/*db*+G-CSF; ^†^ *p* < 0.05 vs *db*/*db*+*HGF* and *db*/*db*+*HGF*+G-CSF; ***p < 0.05 vs *db* ^/−^, *db*/*db*+G-CSF, *db*/*db*+*HGF* and *db*/*db*+*HGF*+G-CSF (Kruskal–Wallis and Mann–Whitney *U* test)

Baseline and post-intervention functional variables are summarised in Table 1. Glucose levels remained similar in diabetic-treated and non-treated groups, suggesting that neither *HGF* nor G-CSF treatments have any effect on hyperglycaemia. Nevertheless, *HGF* gene therapy and/or G-CSF administration halted progression of albuminuria. We further assessed whether these therapies had a role in the progression of glomerulosclerosis (Fig. 2a, b). The animals receiving *HGF* either alone or in combination with G-CSF showed significant reduction in both collagen IV and fibronectin glomerular deposition. Isolated G-CSF therapy was not associated with any significant reduction of glomerulosclerosis. Accordingly, *HGF* gene therapy rather than G-CSF reduced the *Tgf-β/Hgf* mRNA ratio in renal tissue (Fig. 3).

### Hematopoietic stem cell mobilisation and SDF-1 renal production induced by *HGF* and G-CSF resulted in increased numbers of EGFP^+^ cells in the diabetic kidney

**Fig. 4:**
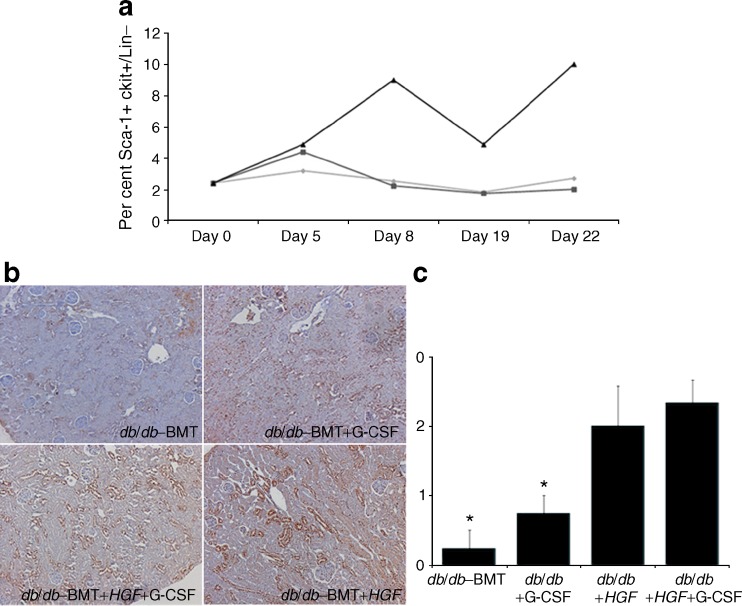
HSC mobilisation and SDF-1 renal expression. **a** Peripheral blood HSC mobilisation. Only the group receiving G-CSF had increased circulating HSC, which was maximal at days 8 and 22 (2 days after each G-CSF injection). *HGF* had no effect on HSC mobilisation. Diamonds, *db*/*db*–BMT; squares, *db*/*db*+*HGF*; triangles, *db*/*db*+*HGF*+G-CSF. **b** SDF-1 renal staining: *db*/*db*–BMT; *db*/*db*+G-CSF; *db*/*db*+*HGF*+G-CSF; and *db*/*db*+*HGF*. Animals treated with *HGF* displayed higher production of SDF-1 in comparison with those without *HGF* treatment. **c** Graph illustrating the mean values of SDF-1 production in different treatment groups. *p < 0.05 vs *db*/*db*+*HGF* and *db*/*db*–BMT+*HGF*+G-CSF (Mann–Whitney *U* test)

**Fig. 5:**
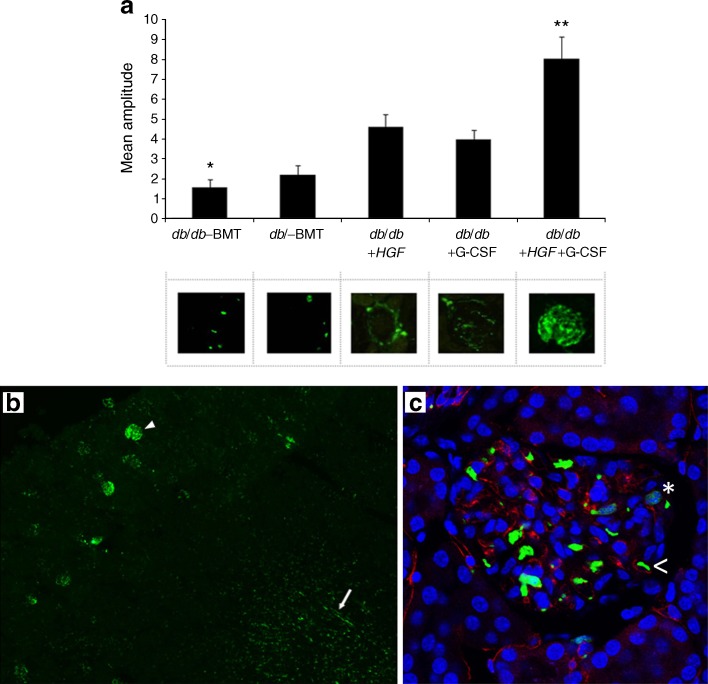
Evaluation of renal EGFP^+^ cells. **a** Mean values of automatic EGFP quantification in renal tissue. *p < 0.02 vs *db*/*db*+G-CSF and *db*/*db*+*HGF*; **p < 0.01 vs *db*/*db*–BMT, *db*/*db*+G-CSF and *db*/*db*+*HGF* (Mann–Whitney *U* test). **b** Representative sample of renal tissue from *db*/*db*+*HGF*+G-CSF group (×40) showing cortical (arrowhead) as well as medullar (arrow) EGFP^+^ cells. **c** Glomerular detail (×630) using CD34 staining (red) in order to identify endothelial cells. The asterisk shows a glomerular extravascular EGFP^+^ cell and the arrowhead shows a circulating EGFP^+^ cell

We analysed whether HGF induced HSC mobilisation into peripheral blood (Fig. 4a). No mobilisation was observed when animals received *HGF* (*db*/*db*–BMT+*HGF*). The addition of G-CSF to these animals significantly increased the number of circulating HSCs. We found *HGF* supplementation was associated with enhanced SDF-1 production in renal tissue (Fig. 4b, c). However, mobilisation of bone marrow cells by administration of G-CSF led to an increased number of these cells in the kidney, as shown in Fig. 5. Administration of *HGF* also led to an increase in renal EGFP^+^ fluorescence, conceivably because of its ability to induce SDF-1 production in renal tissue. The combination of *HGF* gene therapy with HSC mobilisation by G-CSF robustly increased the amount of bone-marrow-derived EGFP^+^ cells in the diabetic kidney (Fig. 5a). We found bone-marrow-derived cells in the renal cortex and medulla (Fig. 5b). Some of these EGFP^+^ cells were localised in diabetic glomeruli (Fig. 5c).

### Renal EGFP^+^ cells are mainly macrophages

**Fig. 6:**
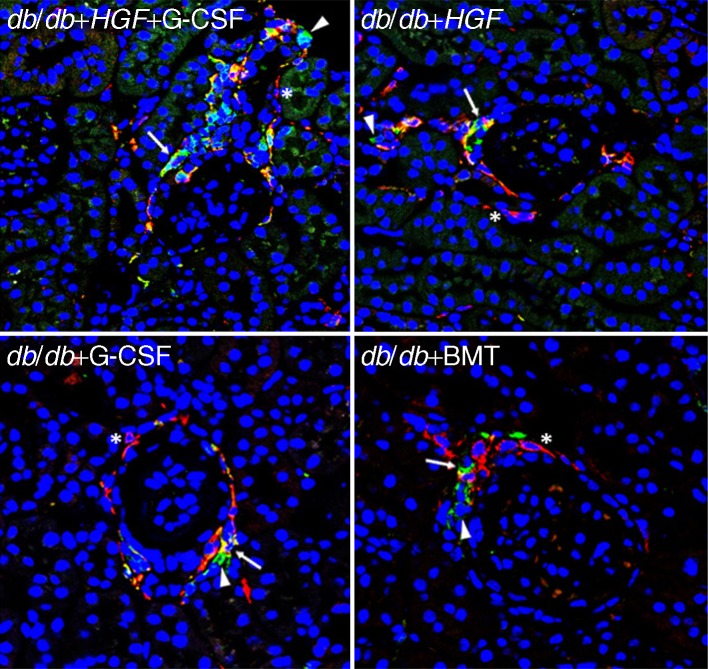
Renal EGFP^+^ cells are mainly macrophages. The arrows show macrophages stained with F4/80 (in red), which co-express EGFP (in green) becoming yellow and therefore suggesting a bone marrow origin. Arrowheads show EGFP^+^ cells (in green). Asterisks show some macrophages stained with F4/80 (in red) that do not co-express EGFP (×630)

There were very few mesenchymal stem cells and all of them were EGFP^−^ (data not shown). Nevertheless, the majority of EGFP^+^ cells located in the kidney expressed the macrophage marker F4/80 (Fig. 6). However, there were also a small number of EGFP^−^ macrophages. Macrophages were mainly localised around the glomeruli and in the interstitial area. Nearly all of the F4/80^+^ cells were EPGP^+^, which means these cells were bone-marrow-derived macrophages.

### HGF gene therapy reduces the inflammatory milieu and enhances the proportion of M2 glomerular macrophages

**Fig. 7:**
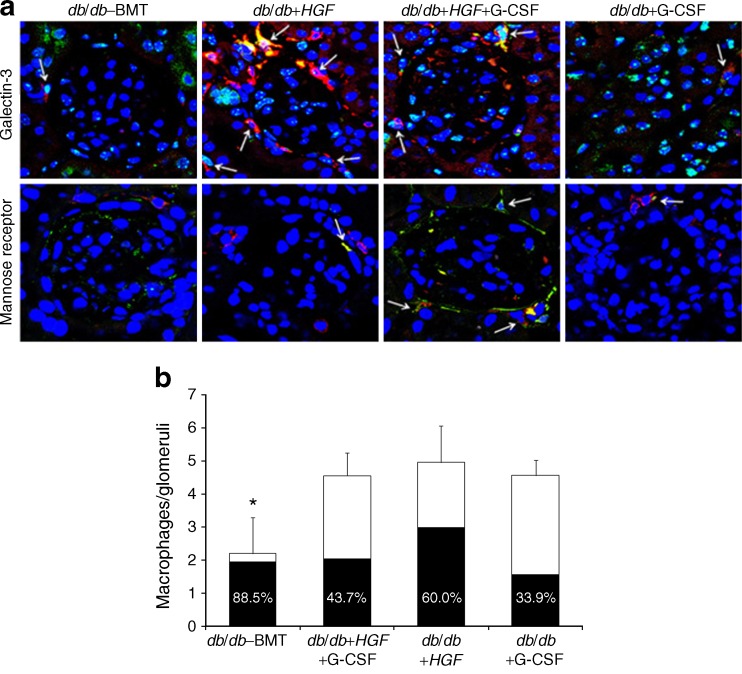
Evaluation of glomerular macrophages expressing M2 markers. **a** Macrophages are stained with F4/80 (in red) and nuclear M2 marker galectin-3 (green), or transmembrane M2 marker mannose receptor (green). Co-staining of both macrophages and M2 markers shows as yellow (arrows) (×630). **b** Number of macrophages per glomeruli. *HGF* gene therapy increased the presence of glomerular macrophages (*p = 0.05, *db*/*db*–BMT vs *db*/*db*–*HGF*), the majority of them (60.0%) expressing M2 markers. Each bar represents the mean number of macrophages F4/80^+^ per glomeruli; the black shading in the bars indicates the percentage of the total macrophages that are M2 macrophages

In order to test whether HGF modifies the inflammatory response we measured serum levels of some Th1, Th2 and pro-inflammatory cytokines in *HGF*-treated groups (Table 2). *HGF* alone and in combination with G-CSF significantly reduced IL-6 and MCP-1. There was also a trend to reduce Th1 cytokines (TNF-α, IFN-γ, IL-12p70) and to enhance some Th2 cytokines (IL-4, IL-10), although these relationships did not reach statistical significance. We evaluated the expression of M2 markers in glomerular macrophages by confocal microscopy (Fig. 7). In agreement with the results shown in Fig. 5, *HGF*, G-CSF and the combination of both were associated with an increased number of glomerular macrophages. Nevertheless, the percentage of macrophages expressing the M2 markers galectin-3 and mannose receptor was nearly double in animals treated with *HGF* vs those receiving G-CSF alone (Fig. 7b). In diabetic non-treated mice, glomerular macrophages were seldom encountered, although a high proportion of macrophages expressed M2 markers.

**Table 2:** Serum levels of Th1, Th2 and pro-inflammatory cytokines in *HGF*-treated animals

Group	TNF-α	IFN-γ	IL-12p70	IL-5	IL-4	IL-10	IL-6	MCP-1
*db*/*db*–BMT	6.5 ± 1.6*	0.6 ± 0.3	20 ± 10^†^	0.5 ± 0.2	0.1 ± 0.0	1.1 ± 0.6	3.9 ± 1.0^‡^	29 ± 5.4^†^
*db* ^/−^	3.4 ± 0.7	0.4 ± 0.3	2.1 ± 2.0	0.2 ± 0.1	0.4 ± 0.3	2.0 ± 0.7	0.4 ± 0.2	17 ± 2.2
*db*/*db*+*HGF*	2.9 ± 0.2	0.2 ± 0.1	6.1 ± 2.2	0.3 ± 0.3	0.7 ± 0.5	3.2 ± 1.0	1.0 ± 0.4	16 ± 1.9
*db*/*db*+*HGF*+G-CSF	1.5 ± 0.2	0.1 ± 0.1	6.2 ± 3.1	0.6 ± 0.4	0.7 ± 0.6	2.5 ± 1.0	0.8 ± 0.3	16 ± 2.0
*p* value	0.08	0.5	0.1	0.5	0.5	0.3	0.01	0.09

*p < 0.05 *db/db*–BMT vs *db*/*db*+HGF+GSF; ^†^ *p* < 0.05 *db*/*db*–BMT vs *db*/–; ^‡^ *p* < 0.05 *db/db*–BMT vs *db/*–, *db*/*db*+HGF and *db*/*db*+*HGF*+GSF (Kruskal–Wallis and Mann–Whitney *U* tests)

### *HGF* gene therapy is associated with the presence of Bowman’s capsule epithelial cells producing EGFP and preservation of podocytes

**Fig. 8:**
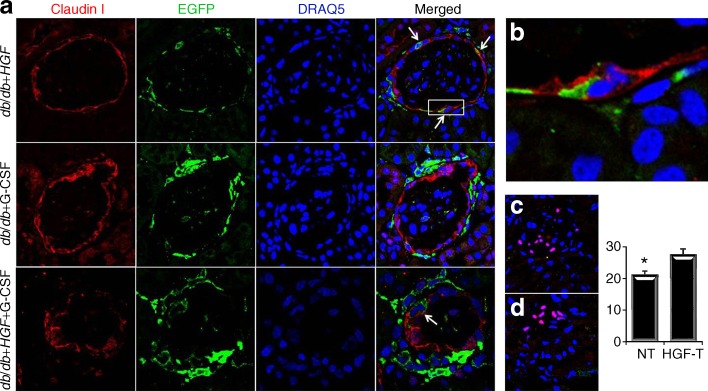
HGF is associated with some parietal glomerular epithelial cells expressing EGFP and higher podocyte number. **a** Only mice receiving *HGF* gene therapy show glomerular parietal epithelial cells that express EGFP. Arrows in merged column are pointing at cells co-expressing claudin-I (red) and EGFP (green). **b** Amplified parietal epithelial cells expressing EGFP. Nuclear podocyte staining with WT-1 (in red) in *HGF*-treated (**c**) and *HGF*-non-treated mice (**d**). Nuclei are stained in blue with DRAQ5. Pink colour is the result of merged red (WT-1) and blue (DRAQ5). **e** Histogram showing the percentage of podocytes in *HGF*-treated and *HGF*-non-treated (‘non-treated’) animals (*p = 0.002, Student’s *t* test)

There was no co-expression of mesangial, endothelial and podocyte markers. Nevertheless, we found a small number (fewer than 0.5%) of Bowman’s capsule parietal epithelial cells (PECs) that were also EGFP^+^. Interestingly, this only happened in mice that received *HGF* gene therapy either alone or in combination with G-CSF (Fig. 8a, b). Accordingly, we found the percentage of podocytes was well preserved in HGF-treated compared with non-treated diabetic animals (Fig. 8c–e).

## Discussion

In the present study we show that *HGF* gene therapy enhances renal expression of SDF-1 and is also associated with an increasing number of bone-marrow-derived cells getting into the injured diabetic kidney. These cells are mainly macrophages, which may fuse with resident epithelial cells from the Bowman’s capsule and participate in renal repair and regeneration.

We have created chimeric diabetic *db*/*db* mice by BMT with bone marrow from donor transgenic EGFP^+^ mice. Thus, we obtained a diabetic mouse model with easily identifiable bone-marrow-derived cells in blood and peripheral tissues. The wild-type C57BL/6J mice express leptin receptor and may have distinct properties from their *db*/*db* counterparts. Despite bone marrow EGFP^+^ cells came from the wild type, we proved BMT was not associated with any significant modification of diabetic nephropathy, as there were no differences between *db*/*db* and *db*/*db*–EGFP^+^ chimeric mice regarding hyperglycaemia, body weight or albuminuria. We were also able to reproduce previous findings from our group concerning the functional and histological benefits of *HGF* gene therapy on diabetic nephropathy in this model. Thereafter, in this work we have investigated whether some bone-marrow-derived cells are in some way involved in HGF-mediated renal repair.

We found that both HGF and G-CSF increased the presence of bone-marrow-derived cells in the kidney, especially surrounding the glomeruli, by different and unconnected mechanisms. We observed that G-CSF consistently induced HSC mobilisation in peripheral blood, whereas *HGF* had no effect on HSC mobilisation. Nevertheless, *HGF* gene therapy enhanced SDF-1 production in renal tissue, which is a major ligand for the C-X-C chemokine receptor 4 (CXCR4) molecule expressed on HSCs [23]. Enhanced SDF-1 in renal tissue has been reported to induce homing of CXCR4^+^ cells to the kidney after ischaemic injury [24]. Accordingly, *HGF* enhanced renal SDF-1 and led to an increased number of EGFP^+^ cells getting into the kidney, despite the lack of induced HSC mobilisation. The combination of *HGF* gene therapy with HSC mobilisation by G-CSF increased the amount of bone-marrow-derived EGFP^+^ cells in the diabetic kidney since G-CSF induced their mobilisation and HGF induced their recruitment. The cells located around the glomeruli were mainly macrophages. In kidney disease, it has been suggested that monocyte-derived macrophages infiltrating the kidney cause injury and fibrosis in renal tissue [25], but can also facilitate kidney repair and regeneration, depending on the renal milieu [26]. Despite finding similar amounts of macrophages in diabetic kidneys from mice treated with *HGF* or G-GSF, we observed consistent histological benefit only in those receiving *HGF* gene therapy. The concomitant administration of G-CSF in *HGF*-treated animals did not provide any additional histological improvement. We have previously described how HGF inhibits pro-inflammatory cytokine expression via inhibition of nuclear factor κB signalling [26]. In agreement, we found *HGF* gene therapy reduced pro-inflammatory cytokines and was associated with a relatively high proportion of reparative M2 macrophages in glomeruli. Therefore, our findings suggest *HGF* gene therapy may induce a renal microenvironment that might promote macrophage-mediated renal tissue repair and regeneration.

We tested whether there were any differentiated glomerular cells co-expressing EGFP. There was no co-expression regarding mesangial, endothelial and podocyte markers. Nevertheless, we found a small number of Bowman’s capsule PECs that were also EGFP^+^. Interestingly, this only happened in mice that received *HGF* gene therapy. These highly specialised cells, which cover the glomerular capillary tuft, are crucial for podocyte repair after injury [27]. Accordingly, we found the percentage of podocytes was well preserved in *HGF*-treated compared with non-treated diabetic animals. It has been suggested that some of these PECs are actually renal stem cells that could migrate along the Bowman’s capsule and transition to the tuft to areas of injury and differentiate into podocytes [28]. Sagrinati et al [28] demonstrated that PECs derive from a resident renal population during kidney development rather than from the bone marrow. Our data suggest these PECs expressing EGFP derive from cell fusion. First, we found extensive macrophage infiltrates around the capsule. Second, macrophages have the ability to undergo cell–cell fusion with themselves or other cell types as renal resident cells, particularly in response to inflammatory stimuli [29]. Therefore, our data suggest HGF attracts bone-marrow-derived cells around the renal capsule, and these can fuse with PECs and probably help repair. Altogether, our findings add new knowledge and open new opportunities for inducing renal regeneration in diabetic nephropathy.

## Abbreviations

BMT: Bone marrow transplantation
CKD: Chronic kidney disease
CTGF: Connective tissue growth factor
EGFP: Enhanced green fluorescent protein
G-CSF: Granulocyte-colony stimulating factor
GFP: Green fluorescent protein
HGF: Hepatocyte growth factor
HSC: Haematopoietic stem cell
PBMC: Peripheral blood mononuclear cell
PEC: Parietal epithelial cell
SDF-1: Stromal-cell-derived factor-1
